# Multisystem Inflammatory Syndrome in Children (MIS-C) Post COVID-19 Infection: Diagnosis and Treatment from the Paediatric Intensive Care Unit (PICU) at a Tertiary Hospital in the Kingdom of Bahrain

**DOI:** 10.7759/cureus.50006

**Published:** 2023-12-05

**Authors:** Sayed H Almosawi, Amreen Mustafa, Fareedul A Hassan, Ebrahim Abousher, Jenan S Nasser, Ramaning Loni, Gabriel Fox

**Affiliations:** 1 School of Medicine, Royal College of Surgeons in Ireland, Manama, BHR; 2 Department of Pediatrics, King Hamad University Hospital, Busaiteen, BHR; 3 Medicine, Sandwell and West Birmingham National Health Service (NHS) Trust, Birmingham, GBR

**Keywords:** corticosteroids, ivig, picu, children, mis-c

## Abstract

Background: Post coronavirus disease 2019 (COVID-19) infections, children presented with varying clinical manifestations of a Multisystem Inflammatory Syndrome in Children (MIS-C). The aim of our study is to identify the clinical manifestations upon admission to paediatric intensive care unit (PICU) and to identify how different treatments affected post-treatment outcomes.

Methods: A retrospective cross-sectional study included 11 patients diagnosed with multisystem inflammatory syndrome based on the World Health Organization (WHO) classification and treated by intravenous immunoglobulin (IVIG) with/without corticosteroids.

Results: There were six female and five male patients with an average age of 5.08±4.7 years. Ten had a confirmed positive severe acute respiratory syndrome coronavirus 2 (SARS-CoV-2) serological antibody test or polymerase chain reaction (PCR) test, with one having only contact history. The most common systems affected by MIS-C were gastrointestinal and ophthalmological presentations. All presented with fever and raised inflammatory markers (erythrocyte sedimentation rate, C-reactive protein, procalcitonin, ferritin, d-dimer, and lactate dehydrogenase). There was no statistical difference between both treatment groups. Clinical and inflammatory markers significantly improved in both groups.

Conclusion: This study highlights an improved outcome associated with combination therapy, although there is no statistical significance between both groups.

## Introduction

During the initial phase of the coronavirus disease 2019 (COVID-19) pandemic, it was believed that pediatric patients had less severe COVID-19 presentations compared to adults [1]. In April 2020, at the peak of the pandemic, reports of severe systemic hyperinflammatory conditions emerged in the United Kingdom, with similar clinical patterns observed in several other countries with higher COVID-19 prevalence rates [2]. These observations prompted an investigation into the underlying cause of these atypical Kawasaki-like disease presentations, characterized by clinical features including prominent gastrointestinal symptoms, lymphocytopenia, and cardiovascular implications, which were found to be associated with recent infections of the severe acute respiratory syndrome severe acute respiratory syndrome coronavirus 2 (SARS-CoV-2) [2]. Subsequently, this condition was labelled as Multisystem Inflammatory Syndrome in Children (MIS-C). Due to the novelty of this syndrome, there was no specific treatment guideline for these patients.

Our retrospective cross-sectional study aims to address this gap by examining the impact of therapeutic interventions, specifically the combination therapy of intravenous immunoglobulin (IVIG) and corticosteroids compared to IVIG alone, on outcomes in patients admitted to the paediatric intensive care unit (PICU) with MIS-C associated with a history of SARS-CoV-2 infection. Through this study, we aim to potentially uphold clinical significance by exploring different treatment modalities and identifying potential trends within demographics and clinical profiles to enhance the understanding and management of MIS-C.

## Materials and methods

Ethical considerations

The study was approved by the King Hamad University Hospital’s (KHUH) Institutional Review Board (#22-550). 

Selection of patients and settings

A total of 15 patients were admitted in the PICU with post-COVID-19 MIS-C between April 2020 and January 2023 at KHUH. The following data was recorded: demographic characteristics, comorbidities, clinical profile, contact history and COVID-19 diagnostic information, blood markers, treatments, and course during hospitalization. Each case was categorized as either confirmed MIS-C or not based on the established criteria by the World Health Organization (WHO). A total of 13 patients met the WHO criteria for MIS-C. Of the two excluded, one had no history of confirmed COVID-19 infection and one had other possible etiologies explaining their symptoms. Of those who had MIS-C, two were excluded as they did not receive either IVIG or corticosteroids. The remaining 11 patients were included in this study. In order to provide a more comprehensive description of the patient population, we also categorized the subjects into three distinct age groups: school-going, preschool, and non-school-going. This categorization allowed for a more nuanced analysis of the data, capturing important developmental and environmental factors that may impact the outcomes. By stratifying the sample in this manner, we aim to better capture the heterogeneity within the patient population and ultimately to improve the generalizability of our findings. 

Data processing and statistical analysis 

Statistical analyses were carried out using SPSS software, version 25.0 (IBM Corp., Armonk, NY). A p<0.05 was considered statistically significant. The initial exploration involved descriptive analysis of the population, utilizing frequency distributions. Subsequently, Chi-square tests were performed to explore associations between key variables and clinical outcomes. We investigated how different treatment courses correlated with variables such as gender, age categories, past surgical history, duration of PICU stay, and presence of comorbidities. Our aim was to unveil and compare the significance of these categories in relation to treatment plans, ultimately discerning the most effective treatment pathway for MIS-C patients.

## Results

There were 11 patients, six (54.5%) were female, and five (45.5%) were male. The mean age was 5.08±4.73 years, with a minimum age of 0.42 years and a maximum age of 13 years. Of those included, four (36.3%) had previous comorbidities prior to MIS-C admission in the PICU. All had no past surgical history (Table 1).

**Table 1:** Baseline characteristics of the study sample PICU: Paediatric intensive care unit.

Baseline characteristic	n=11	%
Gender		
Male	5	45.45%
Female	6	54.55%
Age Category		
<2	4	36.36%
3-<6	3	27.27%
6-14	4	36.36%
Age (years)		
Mean ± standard deviation	5.08 ± 4.73	
Interquartile Range (min-max)	12.58 (0.42-13)	
Body mass index (kg/cm2)		
Mean ± standard deviation	19.59 ± 7.73	
Past Surgical History		
Yes	0	0.00%
No	11	100.00%
Duration of PICU Stay		
≤ 7 days	4	36.36%
> 7 days	6	54.55%
Comorbidities		
Yes	4	36.36%
No	7	63.64%
Clinical Profile		
Constitutional	11	100.00%
Fever	11	100.00%
Abnormal Inflammatory Markers	11	100.00%
Eyes	5	45.45%
Ear nose throat	3	27.27%
Respiratory System	2	18.18%
Cardiovascular	2	18.18%
Gastrointestinal	8	72.73%
Genitourinary	1	9.09%
Musculoskeletal	3	27.27%
Skin/Breast	2	18.18%
Neuro	4	36.36%
Endocrinal	0	0.00%
Heme/Lymph	0	0.00%
Allergy/Immunology	0	0.00%
Psychological	0	0.00%
Treatment		
Intravenous immunoglobulin only	3	27.27%
Intravenous immunoglobulin and corticosteroids	8	72.73%

Most (91%) had either a confirmed positive SARS-CoV-2 serological antibody test or a nasopharyngeal reverse transcriptase-polymerase chain reaction (PCR) test. The two most common manifestations were gastrointestinal (72.7%) and ophthalmological (66.1%). Other clinical manifestations included neurological (36.4%), otolaryngological (27.3%), musculoskeletal (27.3%), dermatological (18.2%), cardiovascular (18.2%), respiratory (18.2%), and genitourinary (9.1%). 

In terms of first-line therapy, 73% received IVIG and corticosteroids and 27% received IVIG alone. In comparison to those who received IVIG alone, those who received IVIG and corticosteroids had a more severe initial presentation, with more frequent abnormal inflammatory markers. More patients who received IVIG alone had a history of previous comorbidities. Previous comorbidities had a strong relationship with the type of treatment, with the p-value as 0.027. In patients with PICU admissions for over seven days (n=8) 27.3% received IVIG alone and 72.7% received IVIG and corticosteroids. In patients admitted to the PICU for or less than seven days (n=3), 66.7% were given IVIG and corticosteroids, and 33.3% were given no form of treatment (Table 2).

**Table 2:** Treatment of choice coinciding with observed characteristics ^E^Constitutional, Fever, Abnormal Inflammatory Markers, Endocrinal, haematic, lymphatic, immunological, and psychiatric systems were excluded in this table, albeit recorded in Table 1. ^*^Significant p-value

	Intravenous immunoglobulin only (n=3, 27.3%)	Intravenous immunoglobulin and corticosteroid (n=8, 72.7%)	P value
Gender			0.621
Male	1 (33.3)	4 (50)	
Female	2 (66.7)	4 (50)	
Age category			0.027*
<2	3 (100)	1 (12.5)	
3-<6	0	3 (37.5)	
6-14	0	4 (50)	
Duration of pediatric intensive care unit stay			0.266
≤ 7 days	0	3 (37.5)	
> 7 days	3 (100)	5 (62.5)	
Comorbidities			0.007*
Yes	3 (100)	1 (12.5)	
No	0	7 (87.5)	
Clinical profile^E^			
Eyes	0	5 (62.5)	0.064
Ear, nose, and throat	0	3 (37.5)	0.214
Respiratory	0	2 (25)	0.338
Cardiovascular	0	2 (25)	0.338
Gastrointestinal	3 (100)	5 (62.5)	0.214
Genitourinary	0	1 (12.5)	0.521
Musculoskeletal	0	3 (37.5)	0.214
Skin/Breast	0	2 (25)	0.338
Neuro	1 (12.5)	3 (37.5)	0.898

**Figure 1:**
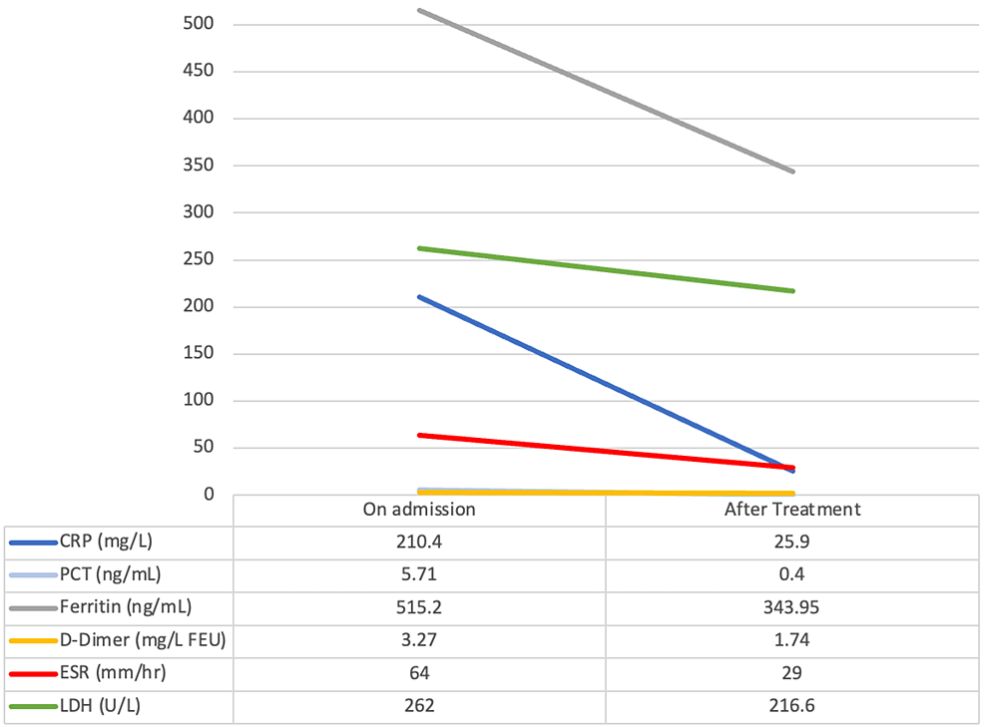
Changes in the medians of abnormal blood markers on admission and after treatment for those treated with IVIG and corticosteroids CRP= C-Reactive Protein; PCT= Procalcitonin; ESR= Erythrocyte Sedimentation Rate; LDH= Lactate Dehydrogenase; IVIG: Intravenous Immunoglobulin.

**Figure 2:**
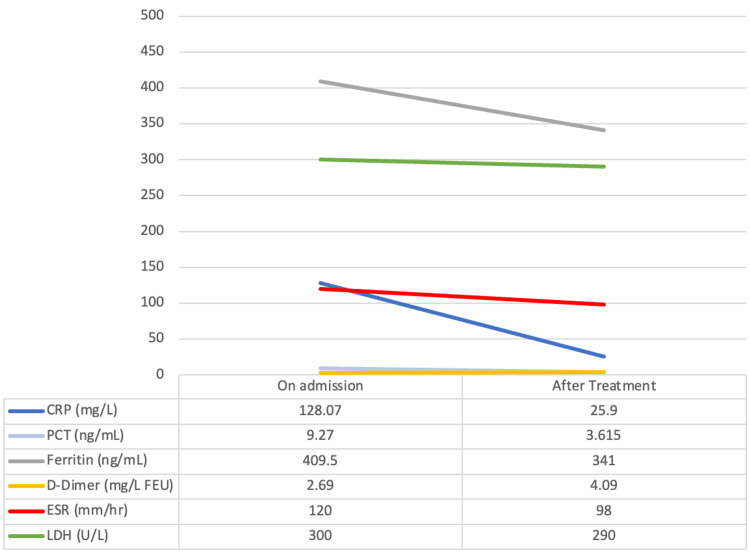
Changes in the medians of abnormal blood markers on admission and after treatment for those treated with IVIG only CRP= C-Reactive Protein; PCT= Procalcitonin; ESR= Erythrocyte Sedimentation Rate; LDH= Lactate Dehydrogenase; IVIG: Intravenous Immunoglobulin.

All presented with a fever and abnormal inflammatory markers, including elevated erythrocyte sedimentation rate (ESR), C-reactive protein (CRP), procalcitonin, ferritin, d-dimer, and lactate dehydrogenase (LDH). Non-parametric independent sample tests were utilized to analyze the distribution of the pre-treatment acute inflammatory markers, which was the same across both groups. The distribution of the post-treatment acute inflammatory markers was the same across both groups (CRP (p=0.776); PCT (p=0.286); Ferritin (p=0.517); D-dimer (p=0.085); ESR (p=1.00); LDH (p=0.548)). Due to the limited sample, medians were utilized to showcase the changes in Abnormal Blood Markers pre- and post-treatment (Figures 1, 2).

## Discussion

In this study, we elaborate on the clinical characteristics of 11 cases of MIS-C, fulfilling WHO criteria for diagnosis. The small study sample was observed in various pediatric intensive care unit (PICU) studies that examined MIS-C [3,4]. These 11 patients were all given treatments while admitted in the PICU, thus aiming at providing clinicians with evidence based practices that are required to treat or reduce the symptoms of MIS-C. Furthermore, it is crucial to note that the initial treatment regimen for all MIS-C cases consisted of IVIG alone 76.4% of the time, according to a recent systematic review [5]. 

In the early stages of understanding and managing MIS-C, the criteria for the indication of corticosteroids was not clearly defined, and the administration of corticosteroids occurred when cases were deemed as too severe. Given the striking similarities in clinical presentation between MIS-C and Kawasaki's disease, physicians initially adopted a treatment approach akin to that used for Kawasaki's disease, combining IVIG and corticosteroids. Over time, as more cases were documented and studied, it became apparent that this treatment strategy yielded positive outcomes for many MIS-C patients. As a result, this approach was tailored to each patient's specific symptom severity, ensuring a more personalized and effective management plan. This pragmatic course of action, combining the knowledge from existing treatments with careful consideration of symptom severity, played a pivotal role in the early management of MIS-C, providing relief and hope to affected children and their families. In our study, physicians initially administered IVIG to all patients with fever and if it resolved within 24 hours, corticosteroid was not administered.

Several studies have reported different outcomes regarding the therapeutic measures for MIS-C treatment. Early administration of IVIG and corticosteroids resulted in shorter patient stays in the PICU [6]; meanwhile, an early combination therapy of IVIG and corticosteroids reduced the likelihood of cardiovascular complications compared to IVIG alone [7]. Our patients did not develop major cardiovascular complications during their admission to the PICU, as corticosteroids were administered rapidly if their fever did not resolve. Furthermore, other studies reported that physicians did not have a general rule of when to administer corticosteroids [8]. This makes our study unique as those with a fever that did not resolve within 24 hours were administered corticosteroids.

The age distribution in our study was 5.08 ± 4.73 years. It is crucial to note that MIS-C can affect individuals across all age groups, particularly those older than five years, with median ages reported between six and 12 years [9]. In studies conducted in the PICU, the mean age was four years [3,10], while in studies in other Western countries, the mean age was six years and older [4,11-13]. MIS-C can affect those over the age of 18 years old also [4,10]. MIS-C may affect people of all ages but has a higher probability of affecting a younger age group.

We found a statistically significant association between age groups and treatment choices (p = 0.027). Those below the age of two were more likely to be treated with IVIG alone, while older patients were more likely to receive a combination therapy of IVIG and corticosteroids. While this group is small and should be interpreted with caution, it suggests that both younger and older patients have better outcomes when treated with IVIG and corticosteroid combination therapy.

The presence of comorbidities also showed a statistically significant association with the choice of treatment (p = 0.007). All those with comorbidities received a single therapy of IVIG, whereas all those without comorbidities received a combination therapy of IVIG and steroids. Although initial studies reported no significant association between underlying comorbidities and the severity of MIS-C, subsequent research has indicated that comorbidities are associated with worsened disease progression [14]. This emphasizes the importance of considering the presence of comorbidities when making treatment decisions for patients with MIS-C. Additionally, it strongly indicates that MIS-C may preferentially affect patients with weaker immune systems.

Obesity was found to be the most common comorbidity [10,11,14]. However, we noticed that the average BMI is 19.59 ± 7.73 kg/cm^2^, indicating that our cohort is in between the underweight and normal range of BMI categories. Obesity may not be indicated in our cohort, but they fall within the borderline normal category, which is also a potential comorbidity. 

Since our patients were admitted to the PICU, the majority of them received a combination therapy, while patients receiving IVIG alone had a longer duration of stay in the PICU. Among those with a PICU stay of seven days or less, none received IVIG alone, while the majority received IVIG and corticosteroids (p=0.266). This finding coincides with many other studies, as one single-center study demonstrated that the implementation of corticosteroids with IVIG resulted in a much shorter PICU stay [14]. Compared to other studies, the length of stay for PICU admissions may lie between five and seven days [3,4,13].

Our study discovered a significant prevalence of gastrointestinal (GI) manifestations in patients with MIS-C, affecting 72.73% of the patients. This aligns with other studies reporting GI as the most targeted system in MIS-C cases [5,8,9,15,16]. The prevalence of GI manifestations may be attributed to a distinctive pathophysiological process in which SARS-CoV-2 antigens increase gut permeability [10]. The distribution of GI involvement was observed across all age groups, making it the most frequently affected individual system. It is worth noting that a previous study found that 50% of patients with GI manifestations were above the age of 10 [9].

In our study, 45.45% of patients had ophthalmological hyperinflammation, with all of them requiring combination therapy of intravenous immunoglobulin and corticosteroid. In two separate studies, ophthalmological involvement was noted to be 49% and 56%, respectively [16,17]. An initial hypothesis arose to explain this phenomenon, stating that SARS-CoV-2-induced endothelial vasculitis of the conjunctival vasculature leads to hemorrhagic non-purulent conjunctivitis [18].

We analyzed various serum markers to assess the inflammatory response in children with MIS-C and we observed that all presented with fever and raised inflammatory markers, such as elevated ESR, CRP, procalcitonin, ferritin, and d-dimer. Medians and means were calculated (Figures 1, 2) in which the markers at admission were high and normalized after treatment in both groups. However, patients treated with combination therapy did present with elevated CRP compared to those treated with IVIG alone. A retrospective study demonstrated similar findings, commenting that ESR, CRP, ferritin, and D-dimer in patients who were administered IVIG and corticosteroids was higher compared to those who were administered IVIG only, with the CRP being a significant value statistically [8]. Another study also documented similar findings. They recorded interleukin (IL)-6 values and found that it was lower in patients who received combination therapy [19]. Similar findings were reported in other studies showing raised inflammatory markers [3,10,20-22].

The findings of our study bear significant implications for future research and clinical practice. However, it's crucial to address the study's limitations. The limited sample size in our study raises concerns about the generalizability of our findings. This constraint underscores the need for future investigations with larger sample sizes and collaborative efforts across multiple centers to strengthen the validity of our conclusions. Furthermore, the retrospective nature of this study introduced inherent limitations as it hindered our ability to measure certain variables and outcomes that could have been more comprehensively assessed in a prospective, controlled study. This limitation underscores the importance of conducting prospective research to capture a more detailed and standardized dataset, especially in the context of a complex syndrome like MIS-C. In addition, the small sample size and retrospective approach restricted our ability to account for variations in treatment regimens among patients. While a randomized controlled trial would be the ideal method for achieving the highest level of accuracy, the severe nature of the syndrome and the challenges posed by its diagnosis in the context of COVID-19 may make it difficult to conduct such a study with a substantial sample size.

## Conclusions

To the best of our knowledge, this study represents the most extensive retrospective investigation of MIS-C conducted in the Kingdom of Bahrain, focusing on patient-specific factors influencing treatment decisions for MIS-C patients in the PICU. MIS-C is a very rare yet serious syndrome. The consensus for managing MIS-C is a combination therapy of IVIG and corticosteroids. Our study highlights different factors which could influence a practitioner’s decision in their choice of treatment regimen. The high prevalence of GI manifestations, the association between comorbidities and treatment choices, and the diverse age distribution highlight the heterogeneity of MIS-C. Despite the small study sample, the results demonstrated that patients treated with IVIG and corticosteroid combination therapy had a better outcome than those treated with IVIG only.
